# Perceptions and preparedness of veterinarians to combat brucellosis through Brucellosis Control Programme in India

**DOI:** 10.14202/vetworld.2020.222-230

**Published:** 2020-02-03

**Authors:** R. Shome, M. Nagalingam, R. Priya, S. Sahay, T. Kalleshamurthy, A. Sharma, R. G. Bambal, H. Rahman, B. R. Shome

**Affiliations:** 1 Indian Council of Agricultural Research-National Institute of Veterinary Epidemiology and Disease Informatics, Yelahanka, Bengaluru, Karnataka, India; 2 Department of Animal Husbandry and Dairying, Ministry of Agriculture, Government of India, Krishi Bhavan, New Delhi, India; 3 International Livestock Research Institute, New Delhi, India

**Keywords:** brucellosis, control program, India, knowledge, veterinary professionals

## Abstract

**Background and Aim:**

Brucellosis caused by bacteria belongs to the genus Brucella is an important zoonosis and constitutes a serious public health hazard worldwide including India. The present study aimed to estimate the knowledge of veterinarians on brucellosis, its public health threat, diagnosis, and vaccination.

**Materials and Methods:**

This cross-sectional study was conducted during 2013-2015 and 453 veterinarians representing 11 states/Union Territories (UT) of India (Assam, Tripura, Meghalaya, Goa, Karnataka, Madhya Pradesh, Uttar Pradesh, Delhi, Jammu and Kashmir, Tamil Nadu, and Punjab) were interviewed using self-administered questionnaire.

**Results:**

Out of 453 veterinarians, 71.74% stated handling of the animals on day-to-day basis and 28.25% were engaged in administration activities. The veterinarians ranked foot-and-mouth disease and brucellosis at the first and fourth ranks among the list of ten economic impacted diseases in the country. A significant association was observed between laboratory confirmation with those who handled brucellosis-suspected cases (p=0.000). Similarly, significant association was noted for the availability of vials/slides (p=0.114), vacutainers (p=0.008), icebox (p=0.103), and refrigerator (p=0.106) for those who preferred laboratory diagnosis. Only 20% of the veterinarians recommended vaccination against bovine brucellosis, and 17% obtained laboratory confirmation for the brucellosis-suspected cases.

**Conclusion:**

The study highlighted the need for awareness programs, laboratory facilities, veterinary doctors, and protective measures for the veterinarians for combating brucellosis through the control program in the country.

## Introduction

In the production of livestock products, India started to attain self-sufficiency from its rapidly growing animal husbandry sector [1]. Still, major parts of livestock economy are affected due to several diseases, and one among those is brucellosis. *Brucella* species are important zoonotic pathogens infecting a wide range of animals causing reproductive disorders. Those affecting domestic livestock are *Brucella abortus* in bovines, camels, and yaks, *Brucella melitensis* causes infection in caprines and ovines *, Brucella ovis* in rams, *Brucella suis* in swine and reindeer, and all these except *B. ovis* infect humans [2-4]. Brucellosis is highly endemic in many states of the country and the highest prevalence has been reported in dairy cattle [5]. The disease was found to be associated with farmworkers, veterinarians, veterinary pharmacists, animal attendants, abattoir workers, and laboratory attendants [6-8]. Many countries such as Canada, the US, Australia, and much of Northern Europe eradicated brucellosis from livestock with the help of lengthy and expensive control programs, but still, it remains endemic in many parts of the world, including India [9]. As far as human brucellosis, many parts of the world have been emerged as new with reports of 500,000 cases yearly, mainly from Mediterranean countries, Central Asia, Arabic Peninsula, India, and Latin America [10]. Brucellosis eradication was made possible by employing the test and slaughter policy elsewhere in the world. However, in India, there are various reasons for its endemicity, namely, ignorance of carrier animals, distress sale of infected animals, ineffective test and slaughter policy in most of the Indian states, lack of effective quarantine, and uncontrolled trans-state migration of animals [11]. The annual median loss indicated for brucellosis in livestock is US $3.4 billion and human brucellosis was estimated to be US $9.07 million (uncertainty interval [95% UI]) with a loss of US $6.39 million among adults and US $2.67 million among children [12,13].

Brucellosis can be prevented over a period of time by one-time vaccination of all eligible female calves. In humans, the use of proper protective measures while handling the infected livestock prevents the disease transmission to a great extent. Although bovine brucellosis has been eradicated in many countries in Europe, Australia, Canada, Israel, Japan, and New Zealand but it is still not controlled in areas such as Africa, the Middle East, and Asia, which is attributed to factors such as dearth of awareness, policies, and resources [9].

Under the 12^th^ 5-year plan of India, Brucellosis Control Programme (B-CP) was introduced in the country to vaccinate bovine calves. For the control program to be successful, the determination of perceptions and preparedness of veterinarians was felt important, and hence, this survey aimed to estimate the knowledge of veterinarians to carry out the activities related to the control program.

## Materials and Methods

### Ethical approval and Informed consent

The study was approved by the ICAR- NIVEDI, India and the authors have taken permission from veterinary doctors to publish the data.

### Sample size and study area

India consists of 29 states and seven Union territories (UTs) and 712 districts with a livestock population of 512 million [14]. This cross-sectional study was conducted during 2013-2015. Under B-CP, providing 1 day brucellosis training to states and UTs was assigned to authors’ institute (Indian Council of Agricultural Research-National Institute of Veterinary Epidemiology and Disease Informatics, Yelahanka, Bengaluru - 560 064, India). The scientists designated in the Brucellosis Control program from the institute organized 1 day brucellosis sensitization training programs at these 11 states/UTs (Jammu and Kashmir, Punjab, Uttar Pradesh, Madhya Pradesh, Delhi, Assam, Meghalaya, Tripura, Tamil Nadu, Goa, and Karnataka), which have covered 299 districts out of 712 districts of the country. The participants in the training were appraised on the current status of brucellosis in the country, zoonotic threat to animal healthcare workers, diagnosis, surveillance, and vaccination drive initiated under B-CP by the Department of Animal Husbandry and Dairying, Government of India before filling up the questionnaire. The states which received the training during 2013-15 were from four regions of the country *viz*., North-Eastern (Assam, Tripura, and Meghalaya); Northern (Jammu and Kashmir, Uttar Pradesh, Delhi, Madhya Pradesh, and Punjab); Western (Goa); and Southern (Tamil Nadu and Karnataka). The participants were selected for the program by the respective state Animal Husbandry Departments ensuring the equal representation from all the districts of the concerned states. The control programs are managed by the designated senior veterinary officer responsible for the district animal husbandry administration activities, and hence, these officers were specifically deputed for the training.

### Questionnaire and data collection

All the veterinarians were explained about the rationale behind the study in the training and informed written consents were obtained before the collection of data. Pretesting of the questionnaire was conducted at the authors’ institute when the veterinarians from all over the country came for the training at one or the other time and the questionnaire was self-administered to the veterinarians before starting the training session. The number of cattle, buffaloes, sheep, and goats handled on a daily basis, number of abortion/infertility/ROP cases handled species wise on daily basis, and brucellosis-suspected cases treated on a daily basis by veterinarians were listed in the questionnaire. The routine practices followed while handling the animals such as use of aprons, gloves, masks, goggles, and protective hygienic measures were collected. For brucellosis diagnosis, availability of basic minimum logistics in veterinary hospitals such as vials, vacutainers, icebox, and refrigerator to store samples was also enlisted in the questionnaire. The list of important diseases was generated during the pretesting of questionnaire as foot-and-mouth disease (FMD), hemorrhagic septicemia (HS), black quarter, peste des petits ruminants (PPR), bluetongue, enterotoxaemia, contagious ecthyma (ORF), brucellosis, parasitic diseases, and leptospirosis. The mean scores obtained from the participants in the scale of 1 to 10 facilitated to designate the ranking for the diseases from the highest to lowest economic importance. The diseases were reshuffled between each participant to avoid scoring biases among the participants. The knowledge of human brucellosis, symptoms observed in their colleagues, and the para-veterinary staff was obtained to interpret their awareness of zoonoses. Veterinarians were asked their opinion on vaccination against brucellosis to understand their view on vaccination policy of the country.

### Statistical analysis

The information collected through the questionnaire from the veterinarians were imported into Microsoft Excel spreadsheet and analyzed in the statistical software IBM^(R)^SPSS 16 Statistics, USA. The Chi-square test and odds ratio were computed to know the significant association within the variables.

## Results

Overall, 453 participants from 11 states/UTs of India were part of the study which include Madhya Pradesh – 17.21% (78/453), Karnataka – 13.90% (63/453), Uttar Pradesh – 12.80% (58/453), Tamil Nadu – 11.47% (52/453), Jammu and Kashmir – 9.05% (41/453), Meghalaya – 7.50% (34/453), Punjab – 6.84% (31/453), Goa – 6.4% (29/453), Delhi – 5.73% (26/453), Assam – 4.63% (21/453), and Tripura – 4.41% (20/453). The study covered 42% of the overall districts of India (299/712) and the senior most veterinarian/s from the state responsible for vaccination and control of disease from the districts was ensured by the state Animal Husbandry Departments for the training. The participation of veterinarians was either one or two and sometimes three from each district except from the states of Assam and Uttar Pradesh (Table-1). This is because senior most veterinary officers were assigned various animal husbandry activities such as implementation of control programs, animal health care, administration, and accounts. Hence, some states have deputed two or three veterinarians for the training so as to have better understanding and coordination in the states while implementing B-CP.

**Table-1:** State and district wise participation of veterinarians in the study.

No.	Regions	States	Number of districts	Number of participants
1.	Northern region	Jammu and Kashmir	22	41
		Punjab	22	31
		Uttar Pradesh	75	58
		Madhya Pradesh	52	78
		Delhi	11	26
2.	Northeastern region	Assam	33	21
		Meghalaya	11	34
		Tripura	8	20
3.	Southern region	Tamil Nadu	33	52
		Karnataka	30	63
4.	Western region	Goa	2	29
Total			299	453

Out of 453 veterinarians, majority of the veterinarians (71.74%) were involved in treating the animals on day-to-day basis compared to officers involved in administrative activities (28.25%). Among 59.16% (268/453) veterinarians who handled abortion/infertility/ROP cases in cattle, 50% happened to be brucellosis-suspected cases. Similarly, 47%, 56%, and 33% of abortion/infertility/ROP cases in buffaloes, sheep, and goats accounted for brucellosis-suspected animals.

**Figure-1:**
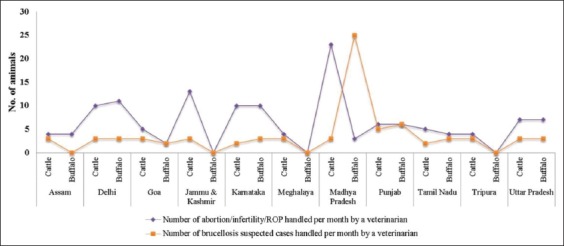
Graph depicting the number of veterinarians handling the abortion/infertility/ROP and brucellosis-suspected cases in bovines.

In a month, 349 cattle, 166 buffalo, 133 sheep, and 323 goats were treated by a veterinarian for various ailments. Among these sick animals, eight animals treated for abortion/ROP/infertility and three among eight of them confirmed to be brucellosis (37.5%). In the case of buffaloes, all the four animals treated for abortion/ROP/infertility were happened to be brucellosis infected. Similarly, majority of animals with a history of abortion/ROP/infertility would be brucellosis- suspected in the case of sheep and goats too. Maximum of five brucellosis-suspected cattle from Punjab, 25 buffaloes from Madhya Pradesh, eight sheep from Jammu and Kashmir, and ten goats from Tripura were handled by a veterinarian per month (Table-2 and Figure-1).

**Table-2:** Number of veterinarians participated and average livestock handled by a veterinarians in states.

	Assam	Delhi	Goa	Jammu and Kashmir	Karnataka	Meghalaya	Madhya Pradesh	Punjab	Tamil Nadu	Tripura	Uttar Pradesh	Total	Percentage
Number of veterinarians participated in training													
Admin	6	3	5	10	31	2	38	6	1	8	18	128	28.25
Handling animals	15	23	24	31	32	32	40	25	51	12	40	325	71.74
Total	21	26	29	41	63	34	78	31	52	20	58	453	
Number of veterinarians handling cases on daily basis													
Cattle	15	23	24	26	32	32	39	25	51	12	39	318	
Buffalo	5	22	23	4	25	13	36	24	29	2	38	221	
Sheep	6	7	2	22	9	4	7	10	43	1	8	119	
Goats	11	19	18	16	21	23	30	11	49	9	33	240	
Number of veterinarians handling abortion/infertility/ROP cases on daily basis													
Cattle	12	20	23	26	30	15	25	23	46	10	38	268	84.27
Buffalo	1	20	17	0	18	0	17	18	8	0	37	136	61.53
Sheep	2	2	0	13	6	1	1	2	12	0	2	41	34.45
Goats	7	13	2	8	19	3	16	7	28	6	15	128	53.33
Number of veterinarians handling brucellosis-suspected cases on daily basis													
Cattle	7	10	6	22	11	6	8	21	17	4	22	134	50.00
Buffalo	0	13	4	0	4	0	9	15	1	0	18	64	47.05
Sheep	0	1	0	12	0	1	2	2	5	0	0	23	56.09
Goats	0	4	0	6	2	2	7	5	8	1	7	42	32.81
Number of cases handled per month by a veterinarian													
Cattle	210	223	249	402	337	196	632	182	551	768	93	349	
Buffalo	42	269	119	53	346	166	364	165	125	75	103	166	
Sheep	90	64	30	263	367	68	99	54	345	30	53	133	
Goats	95	188	57	81	357	159	1701	63	499	247	102	323	
Number of abortion/infertility/ROP handled per month by a veterinarian													
Cattle	4	10	5	13	10	4	23	6	5	4	7	8	2.29
Buffalo	4	11	2	0	10	0	3	6	4	0	7	4	2.40
Sheep	6	4	0	6	15	2	1	2	11	0	7	5	3.75
Goats	4	16	2	2	8	1	9	1	7	1	4	5	1.54
Number of brucellosis-suspected cases handled per month by a veterinarian													
Cattle	3	3	3	3	2	3	3	5	2	3	3	3	
Buffalo	0	3	2	0	3	0	25	6	3	0	3	4	
Sheep	0	1	0	8	0	4	1	1	2	0	0	2	
Goats	0	4	0	2	3	3	1	2	2	10	5	3	

ROP Retention of placenta

**Figure-2:**
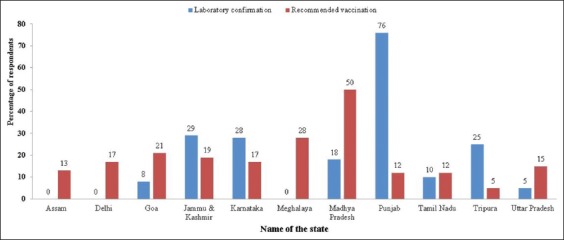
Percentage of respondents obtaining laboratory confirmation and recommended vaccination against brucellosis.

The majority of the veterinarians from Punjab (76%) obtained laboratory confirmation than the other states such as Northern state – Jammu and Kashmir (29%), Eastern state – Tripura (28%), and Southern state – Karnataka (5%). The least number of veterinarians from Uttar Pradesh and Tripura (5%) went for the laboratory confirmation for brucellosis. None of the veterinarians from two North- Eastern states (Assam and Meghalaya) and Delhi opted for laboratory diagnosis for brucellosis-suspected cases (Figure-2).

Very important feedback regarding brucellosis vaccination was sought from the participants, and many veterinarians from Madhya Pradesh recommend vaccination (50%) though only 18% of them obtained the laboratory confirmation. Interestingly, only 12% of the respondents from Punjab recommend brucellosis vaccination contrary to their response on laboratory confirmation (76%). Similar to that of Punjab state, veterinarians from Tripura have given least response in favors of vaccination (5%) though 25% have stated that they do go for laboratory diagnosis. From the overall participants of the training, only 17% obtained the laboratory confirmation for the brucellosis-suspected cases and 20% of the participants recommended vaccination against bovine brucellosis.

**Figure-3:**
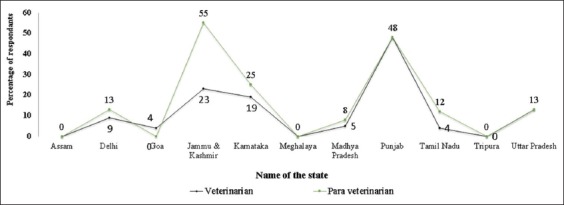
Percentage of respondents noticed the symptoms of brucellosis in humans.

Overall, 17% of the veterinarians who have participated in the training noticed brucellosis symptoms such as undulant fever, joint pain, and orchitis in their subordinate staff (para-veterinarians). Nearly 55%, 48%, and 25% of veterinarians from Jammu and Kashmir, Punjab, and Karnataka states, respectively, have noticed the symptoms of brucellosis in para-veterinarians. The veterinarians provided the feedback that they were aware of their colleague veterinarian friends getting infected with brucellosis and the highest response was from the state of Punjab (48%) followed by Jammu and Kashmir (23%) and Karnataka (19%). Only few from Goa and Tamil Nadu (4%) have noticed brucellosis symptoms in their colleagues. None of the participated veterinarians from Assam, Meghalaya, and Tripura either heard or noticed symptoms of brucellosis among veterinarians and para-veterinarians (Figure-3).

**Figure-4:**
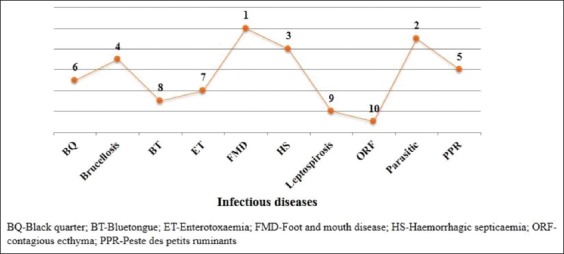
Ranking of infectious animal diseases based on the feedback from the respondents [Ranking from higher (1) to lower economic importance (10)].

**Figure-5:**
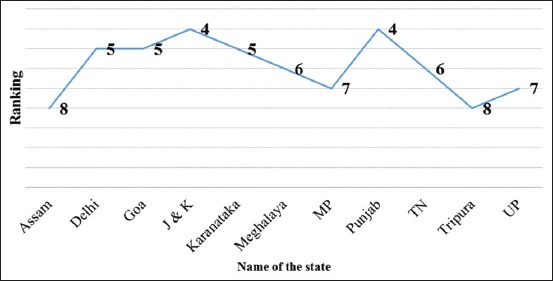
Ranking of brucellosis in different states based on the feedback from the respondents [Ranking from higher (1) to lower economic importance (10)].

Based on their perceptions, the veterinarians ranked FMD at first, followed by the impact of parasitic disease at second, HS and brucellosis at the 3^rd^ and 4^th^ ranks, respectively. High economic impact responses on the livestock due to brucellosis were obtained from Punjab and Jammu and Kashmir states, whereas respondents from Assam and Tripura states have given the least score (Figures-4 and 5). The respondents from other states such as Delhi, Goa, and Karnataka ranked brucellosis on 5^th^.

The laboratory confirmation obtained for brucellosis was significantly associated with those who handled the brucellosis-suspected cases (p=0.000) than those who handled the abortion/infertility/ROP cases (p=0.588). Similarly, significant association to the availability of vials/slides (p=0.114), vacutainers (p=0.008), icebox (p=0.103), and refrigerator (p=0.106) to laboratory confirmation was noted. A significant proportion of veterinarians who obtained laboratory confirmation have recommended vaccination against brucellosis in livestock (p=0.187) (Table-3). Similarly, significant number of veterinarians who were using the gloves while handling abortion did not notice any symptoms of brucellosis (OR = 0.485; 95% CI = 0.205-1.147) and those who used protective hygienic measures (OR = 0.407; 95% CI = 0.162-1.022 (Table-4).

**Table-3:** Association between laboratory confirmation to handling by different laboratory essentials and vaccination recommendations.

		Laboratory confirmation obtained			Chi-square tests value	p-value

		Yes	No	Total		
Handling abortion/infertility/ROP cases	Yes	50	233	283	0.293	0.588
	No	6	36	42		
	Total	56	269	325		
Handling brucellosis-suspected cases	Yes	42	108	149	20.412	0.000**
	No	15	161	176		
	Total	56	269	325		
Vials/slides	Yes	45	188	233	2.503	0.114^$^
	No	11	81	92		
	Total	56	269	325		
Vacutainer	Yes	33	107	140	6.933	0.008**
	No	23	162	185		
	Total	56	269	325		
Icebox	Yes	47	198	245	2.662	0.103^$^
	No	9	71	80		
	Total	56	269	325		
Refrigerator	Yes	44	182	226	2.606	0.106^$^
	No	12	87	99		
	Total	56	269	325		
Vaccination recommended	Yes	38	157	195	1.740	0.187^$^
	No	18	112	130		
	Total	56	269	325		

** 1% level of significance;

$ 20% level of significance

**Table-4:** Risk association between symptoms noticed in veterinarians with the usage of protective measures while handling abortions.

		Symptom noticed			Odds ratio	95% confidence interval	
	
		Yes	No	Total		Lower	Upper
Aprons	Yes	30	229	259	1.104	0.462	2.638
	No	7	59	66			
Gloves	Yes	29	254	283	0.485	0.205	1.147
	No	8	34	42			
Masks	Yes	17	114	131	1.297	0.652	2.582
	No	20	174	194			
Goggles	Yes	12	65	77	1.647	0.784	3.457
	No	25	223	248			
Protective hygienic measures	Yes	30	263	293	0.407	0.162	1.022
	No	7	25	32			

## Discussion

The five biggest states with the highest number of districts in the country are Uttar Pradesh, Madhya Pradesh, Tamil Nadu, Assam, and Karnataka whereas Goa has the least number of districts. One veterinarian per district was ensured for the training program from eight states and one UT except from the states of Uttar Pradesh and Assam. The lack of participation of veterinarians from each district from these two states might be due to large surface area of Uttar Pradesh and inaccessible hilly terrain of Assam state, probably posing difficulty in movement and communication.

The proportion between the number of brucel­losis suspected cases and the number of abortion/Infertility/ROP cases handled by veterinarian is directly related to the evidence of brucellosis in livestock. It is well established fact that brucellosis causes reproductive disorders such as abortion [15], metritis/endometritis [16], retention of placenta (ROP)[17], and repeat breeding/infertility [18]. Overall, among the species, brucellosis-suspected cases were higher in sheep 56% than bovines (cattle – 50% and buffaloes – 47%) and goats (33%). This was justified from the nationwide seroprevalence survey confirming the overall seroprevalence of 7.9% sheep and 2.20% goats from different states of the country [11]. Similarly, high seroprevalence of brucellosis in sheep (8.7) compared to goats (5.8), has been reported from surveillance studies conducted in 12 states of the country [19]. It implies that the highest brucellosis-suspected cases handled by veterinarians is in accordance with the higher seroprevalence reports emerged out during the years 2002 to 2016 [ 20, 21].

Brucellosis-suspected cases routinely attended by veterinarians depend on the dominance of livestock species or high prevalence status of the disease in the region. Maximum of five cattle from Punjab, 25 buffaloes from Madhya Pradesh, eight sheep from Jammu and Kashmir, and ten goats from Tripura were treated for brucellosis-suspected cases by veterinarians in a month. In Punjab, the higher percentage of veterinarians were handling brucellosis-suspected cases, which is in agreement with high seroprevalence estimates for livestock ranging from 7.54% to 26.60% reported over the years [20-22]. Similarly, Madhya Pradesh is one of the top five states having sizeable buffalo population, and hence, veterinarians might be handling the highest brucellosis-suspected buffaloes. Similarly, sizeable population of sheep from Jammu and Kashmir and goats from Tripura were brucellosis-suspected which represents regional importance of the livestock species and their dense population in the region.

Most of the veterinarians were handling brucellosis-suspected cases preferred for laboratory diagnosis. In other words,, the laboratory confirmation was carried out because of availability of basic laboratory essentials such as vials/slides, vacutainers, icebox, and refrigerator for the collection and storage of samples for diagnostic purposes. Thus, obtaining the laboratory confirmation can be attributed to the availability of the diagnostic facilities. Since, Punjab stands first among all the states in obtaining laboratory confirmation, it can be concluded that Punjab has better facilities for diagnosis followed by Jammu and Kashmir (29%), Tripura (28%), and Karnataka (25%) states. The laboratory confirmation obtained for brucellosis was significantly associated with availability of vials/slides (p=0.114), vacutainers (p=0.008), icebox (p=0.103), and refrigerator (p=0.106).

From Madhya Pradesh state, 50% of the doctors were in favor of vaccination and the least response was obtained from the states of Punjab and Tripura. The reasons stated were shortage of doctors compared to the huge animal population or priority of vaccinating other livestock diseases such as FMD, HS, PPR, rabies, and Anthrax. Most importantly, the veterinarians perceived that live *Brucella* vaccine is biohazardous to vaccinators, and hence, their response for vaccination was poor. A significant proportion of veterinarians who obtained laboratory confirmation have recommended vaccination against brucellosis in livestock (p=0.187), indicating better awareness.

In the present study, the significant differences could not be figured out for the veterinarians who were using the gloves while handling abortions [OR = 0.485 (0.205-1.147)] and those following protective hygienic measures [OR = 0.407 (0.162-1.022)]. The majority of the veterinarians did not use protective equipment regularly, probably due to the unavailability. In some areas, the protective equipment was available, but doctors were reluctant to use, and the reason given was mere negligence or adapted to routine practice of not using protective measures. Among the protective measures, the gloves were being used comparatively well than the mask and eyewear as they did not consider inhalation and conjunctival routes to be important.

Brucellosis in veterinarians and para-veterinarians may give us a clue about the prevalence of human brucellosis due to occupational exposure. Humans can get the disease through the consumption of raw milk and raw milk products from infected animals and through direct handling of contaminated materials from infected animals, specifically aborted fetuses, fetal membranes, and vaginal secretions [23]. Many veterinarians from Punjab were aware or noticed the symptoms of brucellosis either in veterinarians or para-veterinarians. This is well correlated to the high seroprevalence of human brucellosis (26.6%) reported from Punjab [24] which indicating better knowledge and attitude levels of veterinarians in higher brucellosis prevalence states . Similarly, brucellosis knowledge, attitude, and practice index of 160 veterinarians across four states was significantly correlated to the higher brucellosis prevalence states, implying that good knowledge of veterinarians may be due to the endemicity of the disease [25]. There is a need for imparting training to upgrade the knowledge and to build a positive attitude among veterinarians of other states in the country.

The veterinarians ranked FMD at the first and brucellosis at the fourth rank among the list of ten economic impacted diseases in the country. This perception of veterinarians is justified as per the recent reports, the annual median loss for FMD to be US$ 3322.04 million [26] and brucellosis estimated to be US $ 3400 million [12]. Although brucellosis is ranked at the fourth position among the top ten important diseases, the indirect economic impact of brucellosis is far greater than other diseases such as HS and parasitic diseases. Hence, the implementation of national policy for the control of brucellosis is very essential though opinions and perceptions did not favor vaccination.

The present control program is restricted to only bovines, and small ruminants are left out of the program. The control of bovine brucellosis may result in the surge of infection in other species, including wild animals [18]. Hence, compulsory vaccination of all female bovine and small ruminant population will ensure control of brucellosis in the country based on feedback from voluntary participation of veterinarians rather than structural sampling.

## Conclusion

The study highlights gaps between the knowledge, perception, lack of facilities, and preparedness among the veterinarians in different states for meaningful prevention and control of brucellosis in the country. Vaccination against brucellosis is the best way to control brucellosis and in the study,only 20% of the veterinarians recommended vaccination against bovine brucellosis. Hence, we strongly recommend the following: Cooperation between the National (Ministry of Animal Husbandry, Dairying, and fisheries, Government of India) and State Departments (State Animal Husbandry) to work in unison to raise awareness towards brucellosis, to establish joint surveillance systems, implementing guidelines for movement of animals, high standard of hygiene and increasing the vaccination coverage for two to three decades to control the disease on war footing. The issues such as raising the awareness level among veterinarians, providing laboratory essentials for diagnosis of brucellosis, compulsory use of protective measures, and compulsory vaccination policy are the requirement of national policy.

## Authors’ Contributions

RS and MN designed the questionnaire, conducted the training, and collected the data. RP, SS, and TK entered and analyzed the data. RS, TK, and RP drafted and finalized the manuscript. RGB, AS, HR, and BRS supervised the funding, training schedules, and necessary permission from the states. All authors read and approved the final manuscript.
